# Using Electronic Health Data to Deliver an Adaptive Online Learning Solution to Emergency Trainees: Mixed Methods Pilot Study

**DOI:** 10.2196/65287

**Published:** 2025-12-17

**Authors:** Anna Janssen, Andrew Coggins, James Tadros, Deleana Quinn, Amith Shetty, Tim Shaw

**Affiliations:** 1 Faculty of Medicine and Health, The University of Sydney, Charles Perkins Centre D17, Sydney, 2006, Australia, 61 2 9036 9406; 2 Western Sydney Local Health District, Sydney, Australia; 3 NSW Ministry of Health, Sydney, Australia

**Keywords:** electronic health records, electronic medical records, digital health, emergency care, health professional education, health informatics, practice analytics, EMR, health data, online learning, pilot study, trainee, clinical activities, feasibility, acceptability, online microlearning, adaptive algorithm, adaptive solutions, adaptive program

## Abstract

**Background:**

Electronic medical records (EMRs) are a potentially rich source of information on an individual’s health care providers’ clinical activities. These data provide an opportunity to tailor web-based learning for health care providers to align closely with their practice. There is increasing interest in the use of EMR data to understand performance and support continuous and targeted education for health care providers.

**Objective:**

This study aims to understand the feasibility and acceptability of harnessing EMR data to adaptively deliver a web-based learning program to early-career physicians.

**Methods:**

The intervention consisted of a microlearning program where content was adaptively delivered using an algorithm input with EMR data. The microlearning program content consisted of a library of questions covering topics related to best practice management of common emergency department presentations. Study participants were early-career physicians undergoing training in emergency care. The study design involved 3 design cycles, which iteratively changed aspects of the adaptive algorithm based on an end-of-cycle evaluation to optimize the intervention. At the end of each cycle, an online survey and analysis of learning platform metrics were used to evaluate the feasibility and acceptability of the program. Within each cycle, participants were recruited and enrolled in the adaptive program for 6 weeks, with new cohorts of participants in each cycle.

**Results:**

Across each cycle, all 75 participants triggered at least 1 question from their EMR data, with the majority triggering 1 question per week. The majority of participants in the study indicated that the online program was engaging and the content felt aligned with clinical practice.

**Conclusions:**

The use of EMR data to deliver an adaptive online learning program for emergency trainees is both feasible and acceptable. However, further research is required on the optimal design of such adaptive solutions to ensure training is closely aligned with clinical practice.

## Introduction

### Background

Medical practitioners engage in a variety of formal and informal training activities to stay up to date on best practices for delivering patient care. Activities undertaken by medical practitioners in the context of professional training can take many forms, including mentorship and consultation with peers, attendance at local seminars and international conferences, journal clubs, and other activities [1]. It has also been observed that medical practitioners dedicate considerable time to undertaking education and training activities. An observational study of physicians on hospital wards found that around 7% of observed tasks involved engaging in education or supervision [2]. Another study found that medical practitioners early in their careers spend close to 2 hours every day engaging in training activities [3].

Digital technologies are increasingly being used to share knowledge and evidence between medical practitioners in the workplace [4]. Online learning as a mechanism for delivering training to the medical profession is also increasingly widespread [5]. Benefits of online learning include making training available when and where individuals would like to access it, potentially enabling more innovative approaches to teaching and making information more easily accessible [6]. In the context of training medical practitioners, flexibility or adaptivity has been emphasized as a major advantage of online education [7]. However, online learning can also have disadvantages, and there is disagreement in the literature as to whether delivering training to medical practitioners online impacts learning outcomes [5]. Another potential issue is that online learning can require more self-discipline and greater time management skills to complete than face-to-face training [8]. This phenomenon may be why online learning has been noted to have high completion rates, with 60% being a high completion rate for many online courses [9].

One strategy for strengthening online learning for medical practitioners could be to align it more closely with clinical practice. It has been noted in the literature that current approaches to medical education disconnect learning activities from practice and care delivery [10]. To date, there is limited research into how online learning could be personalized to align it more strongly with clinical practice. A recent scoping review of secondary use of data from all health information technologies (HITs) identified education as one of 4 key domains for this purpose, but data were used in this way in only 1% of the identified studies [11]. Another scoping review exploring the design of dashboards presenting data from HITs to support reflective practice found that these types of visualization tools were often designed to present data to individual medical practitioners to allow them to reflect on their practice, but such platforms rarely incorporated scaffolds that would support learning or other improvement activities [12].

The field of learning analytics has also explored the significant potential of data collected about learners in online programs for enhancing learning. Learning analytics broadly describes the collection, analysis, and reporting of data about learners and their contexts for the purpose of understanding and optimizing learning and the environments in which it occurs [13]. A recent review of the literature identified personalization as a major focus of learning analytics, particularly in the context of online learning environments where large amounts of data about the learning process are routinely collected [14]. Research into learning analytics in medical education has identified several barriers in this space, including implementation challenges, such as how to collect data to use in learning analytics; data management issues, such as governance and access to appropriate data; and outcomes challenges, such as how data can be used to assess learners, programs, and systems [15]. Furthermore, it has been noted in the wider literature on medical education that when training is delivered digitally, analytics collected about learning progress are a potentially rich source of information for informing evidence-based instruction [7].

In the context of online learning for medical practitioner education, routinely collected electronic health data such as that in electronic medical records (EMRs) may have value for aligning learning with clinical practice. While there is a wide range of HITs used in clinical practice, EMRs are a core technology that have been widely adopted in many health care organizations [16,17]. Data from EMRs have been identified as being useful for understanding the practice patterns of medical practitioners [18] and for supporting formative assessment of early-career physicians in the workplace [19]. There is also literature suggesting that health care providers across a range of disciplines are interested in EMR data being harnessed to inform learning and training activities [20]. To date, there has been limited research undertaken into the use of EMR data to support workplace learning for medical practitioners.

There is a small but growing body of research exploring the intersection between EMR data and health professional learning. A recent study investigated the use of EMR data to support medical practitioners undertaking a learning needs assessment, which was subsequently used as the basis for delivering a customized continuing medical education program for the next year [18]. In this study, medical practitioners were provided an EMR report showing their data compared to other participating practitioners, which they then used to identify personalized learning goals. Another study of this type used data from electronic prescribing systems has also been used to personalize the delivery of letters to prescribers advising them of their compliance with antimicrobial prescribing policy [21]. Researchers have also investigated the use of EMR reports to populate dashboards to educate medical practitioners about their performance. In one such study, a dashboard visualizing EHR reports was made available to medical practitioners for 6 months with the goal of reducing overprescribing of antibiotics [22], though this study was not able to demonstrate a change in practice.

Although interest is increasing in the use of EMR to support training for medical practitioners aligned with clinical practice, the data have some limitations for this application. A considerable limitation is that there can be gaps in the data and that the data are not captured for the primary purpose of education [23], and there are limitations on how these data can be used to understand performance [19]. As has already been highlighted, a further limitation is the relatively few examples in the literature demonstrating how to use EMR data to design this type of personalized education [18,22]. Furthermore, current popular approaches for presenting EMR data to medical practitioners to support reflection lack scaffolds such as self-reflection tools, goal-setting options, or learning plans that could be used to support professional learning [12].

### Problem Statement

Despite the plethora of electronic data being collected by the health system and growing interest in their use to support health professional education, there is a dearth of knowledge on how to design data-driven learning interventions for professional learning. This study aimed to address that gap by investigating the feasibility and acceptability of using EMR data to adaptively deliver an online learning program. In the context of this study, feasibility included answering the following research questions:

- Could data be routinely extracted from an EMR to support data-driven learning?
- Could EMR data be linked to microlearning questions to trigger adaptive delivery to different learners?
- How engaging did learners find the data-driven learning program?

A secondary aim was to understand how to design such a program so that learners felt the content was well aligned with their clinical practice. The study investigated EMR data specifically due to the widespread adoption of this HIT in health care organizations [17] and the recognized use of EMR data for understanding clinical practice patterns [18].

## Methods

### Study Design

The study used 3 design cycles to iterate on the design of the adaptive online learning program. The approach was based on a design-based research approach that has been used in research education contexts to provide a systematic but flexible approach to improv practice through iterative cycles of design, development, and implementation [24]. At the end of each cycle, a mixed methodology was used to evaluate the feasibility and acceptability of the program and refine it in response to learner feedback. Within each cycle, learners were enrolled in the adaptive program for 6 weeks, with new cohorts of learners in each cycle.

### Participants and Study Setting

The study was undertaken within the emergency department at 2 public metropolitan hospitals in Sydney, Australia. The 3 design cycles were run over a 12-month period between December 2018 and December 2019.

Potential participants were early-career physicians who were undergoing postgraduate training. Early-career physicians in Australia are those who have graduated from medical school and have provisional registration to practice but have not undergone enough training to be qualified to be independent practitioners. The first step of the training pathway, typically completed within 2 years of postgraduation from a medical degree, is working as an intern for 12 months. As an intern, early-career physicians undertake 10-week terms to expose them to different clinical environments. A rotation in emergency medical care is always included in this training. After working as an intern, early-career physicians may become residents for an additional year, potentially followed by being a registrar to undertake training for a specialized medical pathway [25].

Inclusion criteria for the study required participants to be interns undergoing their emergency medical care term at the study sites during the recruitment period. Participants were excluded from the study if they were not interns at the study sites or were not undertaking an emergency medical term while the study was recruiting.

All interns at the participating study sites were invited to participate in the study. Participants were recruited via flyers and an online notice with a link to sign up for the study. In design cycle 1, 22 physicians consented to participate; in design cycle 2, 36 physicians consented to participate; and in design cycle 3, 18 physicians consented to participate in the study. No physicians formally withdrew from the study.

### Study Procedures

#### Intervention Design

The online program was delivered using the Qstream microlearning platform [26]. This is an off-the-shelf platform that sends multiple-choice questions to learners via email or a smartphone app. To reinforce a single take-home message, multiple-choice questions provided learners with detailed feedback on why the response they had entered was correct or incorrect. By default, the platform delivered learners a small bundle of 2 to 3 questions at a time, so that it only took a few minutes to respond to the bundle. The platform would then repeat questions a set number of times depending on whether the learner answered incorrectly or correctly. In the intervention, the microlearning platform was modified to alter the way questions were selected for individual learners and the frequency at which they were delivered. The modified functionality was an adaptive algorithm using data extracted from the EMR. Data were extracted from the EMR via a report that was run 2 times each week the adaptive program was running. The adaptive algorithm ingested EMR data related to the cohorts of patients an individual participant had encountered in the previous few days. The algorithm would subsequently tailor the delivery of microlearning questions for each learner based on the patients they had encountered in the reporting period. For example, if a participant had seen a patient with a heart condition, they would be sent a question on best practice for managing this patient group. If a peer of the participant had not seen a patient with a heart condition but had seen a patient presenting with shortness of breath, they would receive a question on best practice for managing patients with this condition. Multimedia Appendix 1 shows an example of the Qstream app interface and one of the cases for this intervention.

Questions were developed by a team of domain experts in emergency care and educational designers. The domain experts developed a curriculum that covered common clinical scenarios encountered in the emergency department that were considered opportunities to improve knowledge and behavior related to the best practice of potential participants. The curriculum and the questions that were built within it were developed based on the domain experts’ understanding of common knowledge gaps of the potential participants. The educational designers undertook a structured review of EMR reports to understand the data that were available to trigger the delivery of curriculum content in a manner that would adapt to an individual participant’s clinical encounters during the intervention. The curriculum and question set were designed to be relevant to improve clinical practice of participants as well as feasible to personalize through accessing EMR data and were developed using an evidence-based approach to developing microlearning questions [27]. The final curriculum consisted of cases on the management of care for patients presenting with the following symptoms: (1) chest pain, (2) abdominal pain (triggered by clustering 2 presenting problem codes), (3) breathlessness, (4) syncope (triggered by clustering 2 presenting problem codes), (5) headache, and (6) fever. A seventh category was added to the curriculum for the third cohort of learners: mental health (triggered by clustering 10 presenting problem codes). A total of 45 questions were developed for the original 6 program categories. Five additional questions were developed for the mental health category made available for the third cohort of learners. Questions were not pilot-tested with representatives from the cohort they were developed for prior to use in the intervention. Table 1 presents an overview of the microlearning program curriculum and questions.

**Table 1.** Overview of the microlearning program, including the topics, the question names, and take-home messages, and the cohort that had access to the library.

Topic and question ID		Take-home message	Cohort
Abdominal pain			Cohorts 1- 3
1		In females of childbearing age, ectopic pregnancy should always be the first consideration and a diagnosis of exclusion regardless of the menstrual and conception history reported.	
2		General supportive measures remain consistent in all resuscitation scenarios, especially in the bleeding patient. Warming patients may help prevent worsening coagulopathy and further bleeding.	
3		Abdominal pain in older people can be a challenging presentation. In contrast to younger patients, a broad range of life-threatening differentials should be considered.	
4		In high-stress settings, cognitive readiness may be enhanced using simple techniques that minimize autonomic hyperarousal.	
5		It is important to explore and investigate the causes of bloody or prolonged diarrhea which in this case could be due colitis. Night time defecation is suggestive of an underlying pathological cause.	
6		Pancreatitis is inflammation of the pancreas and involves activation of proteolytic enzymes that may progress to hemorrhagic necrosis of the pancreatic parenchyma. Different geographic locations may report different incidences of aetiologies but universally ethanol and gallstones are common causes.	
7		Intrarenal calculi are not, on their own, an indication for referral to urology.	
8		Examination of the testes is an essential part of the abdominal examination in young males, even if they do not report testicular pain. Torsion is the diagnosis of exclusion.	
Shortness of breath			Cohorts 1- 3
1		A structured approach to x-ray interpretation is required when reviewing in the ED^a^ because we do not normally have the benefit of a *formal* report from a radiologist. Subtle pneumothorax is an easily missed diagnosis.	
2		BiPAP^b^ NIV^c^ therapy is indicated in a patient with an acute exacerbation of COPD^d^ with a persistent respiratory acidosis despite appropriate initial treatment. A low level of consciousness is not an absolute contraindication to the use of NIV.	
3		The treatment of anaphylaxis requires a dose of intramuscular adrenaline. The EpiPen dose is 0.3 mg. Typically, 0.3 mg to 0.5 mg (1:1000=0.3‐0.5 mLs) is the dose for an adult patient with anaphylaxis.	
4		In a patient with hypoxia and known COPD at risk of CO_2_ retention, apply titrated oxygen first until target saturations are achieved. Positioning the patient is also an important management step.	
5		Patients with underlying cancer are at higher risk of life-threatening sepsis (associated with chemotherapy), pericardial effusions, and PE^e^.	
6		A plain CXR^f^ is practical, easily accessible, and universally indicated for a patient with acute shortness of breath in the ED.	
7		It is important to remember that there are noncardiorespiratory differentials to shortness of breath. Patients with a metabolic acidosis will often present with tachypnoea (Kussmaul respiration).	
Chest pain			Cohorts 1- 3
1		Early diagnosis of AD^g^ requires a high index of suspicion. Blood pressure treatment should target control of both heart rate and the pressure itself.	
2		Chest pain “PLUS” another symptom should trigger the thought, “Could this be a diagnosis of Aortic Dissection?*”* AD may mimic acute MI^h^ (including ECG^i^ findings).	
3		No one factor can reliably rule out ACS^j^ in the ED setting. Pain radiating to the right shoulder or arm is considered more specific for ACS than pain radiating to the left arm.	
4		An ECG should be performed in anyone presenting with chest pain. While well known, S1Q3T3 is uncommon in the setting of PE. Sinus tachycardia and anterior *T* wave inversions are more common.	
5		It is important to consider PE as a differential in patients with cancer. There are scoring tools available to assist you (Wells and PERC being the most commonly used in the ED).	
6		A CXR should be part of your workup for chest pain and shortness of breath. Special care should be paid to looking for pneumothorax and consolidation.	
7		MI, PE, and AD are 3 critical conditions to consider with any patient presenting with chest pain.	
8		AD should be considered in any patient with chest pain with risk factors (eg, hypertension), or chest pain with a concurrent symptom such as neurological deficits.	
Fever			Cohorts 1- 3
1		The presence of severe pain (pain out of proportion to the clinical findings) in an at-risk patient should raise concerns about the diagnosis of necrotising fasciitis.	
2		It is important to note the patient’s allergy to penicillin. Tazocin (piperacillin or tazobactam) is a penicillin, therefore is relatively contraindicated for febrile neutropenia, though it remains first line in guidelines.	
3		Early recognition of sepsis is paramount (new concept of qSOFA^k^ rather than use of nonspecific or sensitive “SIRS^l^” criteria).	
4		Patient is MRSA^m^ colonized so we should consider vancomycin as additional cover (with expert advice). Antibiotics choice should always be judiciously guided by guidelines and ID team support	
5		You must cleanse your hands before and after any patient interaction. Alcohol is generally preferable over soap and water unless the hands are soiled or if *Clostridium difficile* infection is an issue (ie, spores are not necessarily killed by alcohol rub).	
Headache			Cohorts 1- 3
1		In the event of a late (>6 h) presentation with a typical SAH^n^ story, further investigations are required to excluded the diagnosis. All headache cases should be discussed with a senior physician prior to discharge.	
2		If a patient presents with an acute severe headache plus fever and if the CT^o^ is normal, consider a lumbar puncture to exclude meningitis. Consider giving early intravenous antibiotics and antivirals.	
3		If a patient with no history of headaches presents with a sudden onset headache and classic features of SAH, and the CT scan comes back *‘normal’* consider further testing. MRI^p^ or MRA^q^ and specialist review may be warranted under these circumstances.	
4		When a patient presents with a headache, be sure to prescribe analgesia. Patients with papilledema require ED evaluation with referral to both ophthalmology and neurology following neuroimaging.	
5		Over analgesia (especially with paracetamol, codeine, and aspirin) is associated with a paradoxical increase in headache in some patients. While analgesia in the acute setting is a mainstay of our ED management, we should discuss refractory cases with a neurologist and arrange follow-up.	
6		Typically, the CSF^r^ glucose concentration is two-thirds that of the serum glucose concentration.	
7		Temporal arteritis is a sight-threatening condition that is also known as GCA^s^. Jaw claudication is a classic symptom.	
8		Low CSF headache is a distinct and familiar syndrome that is seen most frequently following lumbar puncture. Typically, the headache is orthostatic and significantly improves by lying flat.	
Syncope			Cohorts 1- 3
1		Patients presenting with syncope should be risk-stratified based on their overall history, examination, and a period of observation. Risk scores (eg, San Francisco or the Rose Criteria) may have some use as an adjuvant to your thorough clinical assessment.	
2		An ECG is a very important test in a patient with syncope. It is a mandatory test in the ED for a patient with syncope.	
3		Dosing of NOACs^t^ can be confusing and factors such as age, weight, renal function, and indication may affect dosage. Consultation with hematology and a pharmacist regarding dosing is pertinent.	
4		Vertigo is a common ED presentation. The history given is concerning for a “*central*“ cause of vertigo. Age is an independent risk factor for stroke.	
5		Early defibrillation and commencement of high-quality BLS^u^ are critical to outcomes in cardiac arrest. While not contraindicated, checking for a pulse is no longer specifically recommended in assessing for signs of life or confirming arrest.	
6		Metoclopramide and prochlorperazine both worsen Parkinson disease symptoms, and therefore are contraindicated in those with Parkinson disease. Domperidone is more appropriate for these patients.	
7		If in doubt with a case of “wide complex tachycardia,” call for help and assume the diagnosis is VT^v^ until proven otherwise.	
8		If in doubt with a case of ”wide complex tachycardia,” call for help and assume the diagnosis is VT until proven otherwise.	
Mental health			Cohort 3
1		This is a possible first presentation of schizophrenia with paranoid delusions. The importance of a mental state examination and collateral history is highlighted in this case.	
2		Although there are some variations in practice, current guidelines for parental sedation have droperidol as the most appropriate first-line agent. This should be done with care not to cause further harm to the patient, both through physical and medical means. Droperidol is a commonly used agent in the ED at Westmead Hospital.	
3		Alcohol withdrawal can often present in unusual circumstances such as a change of environment limiting someone’s access to alcohol. The presence of visual hallucinations, confusion, and physical complaints make a purely psychiatric diagnosis less likely.	
4		A person may be treated without consent under two conditions: (1) life-threatening emergencies when a patient lacks “capacity”; and (2) under the Mental Health Act (but only for psychiatric treatments).	
5		There is no such thing as “low risk” in assessing the patient with suicidal tendencies. All patients with mental health issues in the ED have a significantly higher long-term risk of suicide than the general population.	

a ED: emergency department.

b BiPAP: bilevel positive airway pressure.

c NIV: noninvasive ventilation.

d COPD: chronic obstructive pulmonary disease.

ee PE: pulmonary embolism.

f CXR: chest x-ray.

g AD: aortic dissection.

h MI: myocardial infarction.

i ECG: electrocardiogram.

j ACS: acute coronary syndrome.

k qSOFA: quick sequential organ failure assessment.

l SIRS: systemic inflammatory response syndrome.

m MRSA: methicillin-resistant *Staphylococcus aureus*.

n SAH: subarachnoid hemorrhage.

o CT: computed tomography.

p MRI: magnetic resonance imaging.

q MRA: magnetic resonance angiography.

r CSF: cerebrospinal fluid.

s GCA: giant cell arteritis.

t NOAC: nonvitamin K antagonist oral anticoagulant.

u BLS: basic life support

v VT: ventricular tachycardia.

#### Intervention Delivery

During the 6-week intervention period for the online program, a report was manually extracted 2 times each week (Tuesdays and Fridays) from the EMR and fully deidentified patient information. The frequency of extraction was chosen for feasibility reasons as the extraction process was manual and we could not continually extract the data. Only structured data were included in the report as EMR data were only being used to trigger the delivery of questions, not to tailor the content in individual cases received by learners. The deidentified report was then provided to the researchers in .csv format. The EMR report was used to identify if participants had interacted with the relevant patients to populate the adaptive algorithm in the microlearning program. If the EMR data indicated a participant had encountered patient presentations related to the microlearning program, they would “trigger” a question related to managing that type of patient presentation. “Triggering” a question meant that a participant would be allocated a relevant question in the microlearning platform, and it would subsequently be pushed to them via email or the smartphone app to complete. If a participant did not see any clinical presentations that could trigger a question in the reporting period, they did not receive any questions. If participants had seen clinical presentations that could trigger a question, they were enrolled in the relevant question. Questions were pushed to each participant on the same day they were generated, so they received the case within 3 days of seeing the patient that triggered it.

The number of questions participants could receive and the topics in the program were iterated on each cycle in response to analysis of data evaluating that cycle. Participants could be re-enrolled in a question at a later point in the intervention period if they had not previously attempted to respond to it, and the EMR data indicated they had triggered it again. Modifications were made to the delivery of the algorithm for the cases in each cycle. Table 2 summarizes the iterations across all the design cycles.

**Table 2.** Summary of iterations made across the design cycles and explanation of why those modifications were made.

Summary of quantitative data	Design cycle 1	Design cycle 2	Design cycle 3
Iterations undertaken	Cases delivered across 6 presenting problem domains. Participants were assigned up to 3 questions each for each EMR^a^ reporting period. If only 1 patient presentation was encountered by a participant during the EMR reporting period, 3 questions related to that patient presentation would be delivered. If no patient presentations that could trigger a question were encountered during the EMR reporting period, then no questions would be assigned.	Cases delivered across 6 presenting problem domains. Participants were assigned up to 3 questions each for each EMR reporting period. If only 1 patient presentation was encountered by a participant during the EMR reporting period, only 1 question related to that patient presentation would be delivered.	Cases delivered across 7 presenting problem domains. Mental health was added as a new presenting problem.
Justification for iterations	N/A^b^	Reduce the number of questions participants had to respond to in a single bundle to increase course engagement	Increase the perceived alignment of the course content with clinical practice by using a less common, but still frequent patient presentation

a EMR: electronic medical record.

b N/A: not applicable.

During design cycle 1, each participant was assigned 3 questions chosen using the personalization algorithm based on the cases they had attended during the EMR reporting period. If only 1 clinical presentation that could trigger a question was seen by a participant, they would receive 3 different questions on that topic from the question library. For example, in a single EMR report, if a participant had seen only patients with chest pain, they would receive 3 chest pain questions; if that participant had seen 1 patient with chest pain, 1 patient with abdominal pain, and 1 patient with syncope, they would receive 1 question on each topic; if a participant had seen nonrelevant patients, they would receive zero questions.

During design cycle 2, participants who saw relevant clinical presentations in each reporting period were assigned up to 3 questions chosen using the personalization algorithm. They only received 1 question per topic triggered by the EMR data, rather than multiple questions as was the case in design cycle 1. This change was made to reduce the number of questions the participants had to respond to in a single bundle, as analysis of temporal data collected in design cycle 1 indicated some participants were completing all cases in a single bundle at the end of each week. For example, in a single EMR report, if a participant had seen only a patient with chest pain, they would receive 1 question on chest pain; if that participant had seen 1 patient with chest pain and 1 patient with syncope, they would receive 1 question on chest pain and 1 question on syncope; if that participant had seen 1 patient with chest pain, 1 patient with abdominal pain, and 1 patient with syncope, they would receive 1 question on each topic; if a participant had seen no relevant patients, they would receive zero questions.

During the final design cycle, the delivery of questions was the same as in design cycle 2; however, questions related to the topic of mental health were added to the program. Mental health cases were added to see if a less common patient presentation would increase the sense of alignment between the intervention and clinical practice.

Regardless of the design cycle, at the end of each week during an intervention period, all participants in that cycle were unenrolled from any questions they had not responded to. This was done to ensure a large backlog of questions did not accumulate for participants to answer, also to ensure participants were not receiving questions that were not aligned with their recent clinical practice. Participants could also be enrolled in the same question multiple times during the design cycle if they had not attempted it on a previous enrollment and had subsequently been unenrolled. There were two situations in which participants would not be enrolled in a new question even if they had triggered it in the EMR report. The first reason a participant was not enrolled in new questions was if they already had questions they had been enrolled in and had not finished answering. The second reason was because a participant had answered all the questions on that topic correctly and had not triggered any other topics based on the EMR report.

#### Intervention Evaluation

To evaluate the program, a number of data points were collected. EMR reports were used to trigger the delivery of questions during the intervention, as well as to determine the time that had elapsed between seeing a clinical scenario and completing a question. Coupled with metrics from the EMR, metrics captured by the online learning platform were extracted to understand participant engagement with the intervention, participant progress during the program, the number of questions participants were enrolled in that they completed, the accuracy of their responses, and the time that elapsed between being allocated a question and answering it. Finally, a bespoke online survey created by the researchers was disseminated at the end of each design cycle to capture participant feedback on the program. Survey responses were anonymous. The survey consisted of a combination of Likert responses and free-text comments. Structured questions explored how participants accessed the program, the value of clinical data for learning, the relevance of the program content, the course format, and overall experience with the course. Free-text responses allowed participants to provide general comments and feedback on changes to the course. The survey questions were not pilot-tested prior to use in the study.

### Data Analysis

Metrics captured by the online learning platform on participant progress during the program were descriptively analyzed to understand how adaptations to the algorithm influenced participant behaviors. These data, combined with EMR reports, were also analyzed to understand how temporal factors related to the alignment of questions with the participant’s clinical practice influenced engagement with content. Reports extracted from the EMR were analyzed to understand the link between participant test ordering and allocation of questions in the online learning platform.

Structured data from the survey were descriptively analyzed. Unstructured data from free-text survey comments were analyzed to understand participant engagement with the content of the intervention, the online learning platform, and the adaptive component. The content analysis was undertaken by one researcher (AJ) who read all the comments to get a sense of the data. Additional read-throughs of the data were undertaken to code the data and subsequently classify it into categories based on the similarity of themes discussed.

### Ethical Considerations

The study received ethical approval from the Western Sydney Local Health District Human Research Ethics Committee (protocol: 2019/ETH02509). All participants provided written informed consent before agreeing to participate in the study. The researchers complied with informed consent guidelines when necessary and have adhered to local, national, regional, and international law and regulations regarding the protection of personal information, privacy, and human rights. Study data was de-identified prior to analysis. Participants did not receive any compensation for participating in the study.

## Results

### Overview

**Figure 1.**
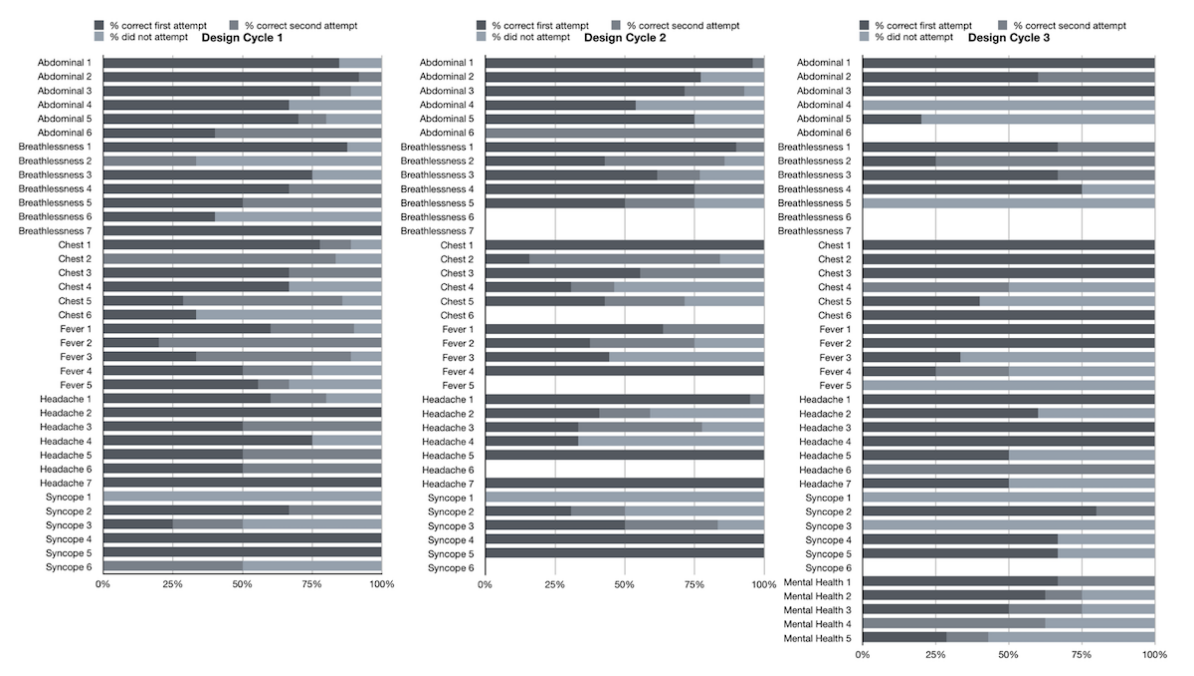
Questions in the program, and the percentage of participants that correctly, incorrectly, or did not respond to for design cycles 1-3.

**Figure 2.**
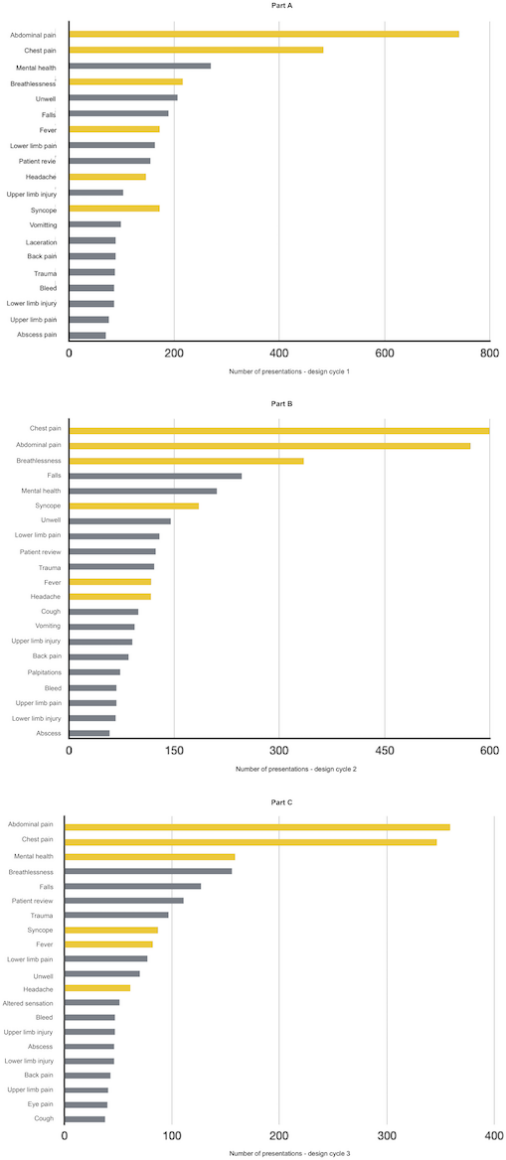
Visualization of the top 20 presenting problems encountered by participants during the intervention period for design cycles 1-3. (A-C) The presentations in design cycles 1-3. The presenting problems that could trigger a question in the online program are highlighted in red.

The following sections present a description of data from each design cycle of the intervention. Table 3 shows an overview of key quantitative data compared across each design cycle, and Table 4 shows an overview of the responses to the online survey across all 3 design cycles. There was a library of 37 questions across 5 topics during design cycles 1 and 2. There was a library of 43 questions covering 6 topics in design cycle 3. If a participant encountered a patient related to one of the topics in the biweekly EMR report (2 times a week) during a design cycle, it would trigger the delivery of a question. Figure 1 shows the question topics in the program and the percentage of participants who correctly, incorrectly, or did not respond to each topic for design cycles 1-3. Figure 2 visualizes the top 20 presenting problems encountered by participants during the intervention period for design cycles 1-3.

**Table 3.** Comparison of key quantitative data points across design cycles 1-3 of the intervention.

Summary of quantitative data	Design cycle 1 (n=21)	Design cycle 2 (n=36)	Design cycle 3 (n=18)
Unique questions in cohort, mean (SD; range)	9.72 (4.62; 3-20)	9.69 (5.79; 1-22)	6.27 (4.93; 1-17)
Range of unique questions in cohort, mean (SD; range)	3‐20 (4.62)	1‐22 (5.79)	1‐17 (4.93)
Participants attempting more than 50% of the questions, n (%)	18 (86)	23 (66)	7 (37)
Total patient presentations (including duplicates) for participant cohort that could have triggered a question, n	1931	4918	2961
Three most common presenting problems encountered by all participants in a cohort that could trigger a case, n	Abdominal pain: 649 presentations Chest pain: 484 presentations Shortness of breath: 215 presentations	Chest pain: 600 presentations, Abdominal pain: 573 presentations Shortness of breath: 335 presentations	Abdominal pain: 357 presentations Mental health: 159 presentations Shortness of breath: 156 presentations
Survey responses, n	9	15	1

**Table 4.** Comparison of survey responses across design cycles 1-3 of the intervention.

Summary of survey responses	Design cycle 1 (n=9), n (%)	Design cycle 2 (n=15), n (%)	Design cycle 3 (n=1), n (%)
Participants who agreed or strongly agreed with the statement that the duration of the course suited their needs	6 (67)	12 (80)	1 (100)
Participants who agreed with the statement they would have like to have received more cases each week.	4 (44)	11 (73)	0 (0)
Participants who agreed or strongly agreed with the statement they found the online program engaging	6 (67)	12 (80)	1 (100)
Participants who agreed or strongly agreed they would recommend the course to a colleague	7 (78)	12 (80)	0 (0)
Participants who agreed or strongly agreed with the statement the program content was realistic	8 (89)	8 (89)	0 (0)
Participants who agreed or strongly agreed with the statement that the content felt aligned with clinical practice.	8 (89)	14 (93)	0 (0)
Participants who agreed or strongly agreed with the statement that the questions in the program felt aligned or linked to their clinical practice.	8 (89)	14 (93)	1 (100)
Participants who agreed or strongly agreed with the statement the program felt engaging because it used clinical data relevant to their practice.	6 (67)	10 (67)	0 (0)
Participants who agreed or strongly agreed with the statement that they would like to see clinical data used to deliver personalized professional development in future.	5 (56)	15 (100)	1 (100)

### Design Cycle 1

There were 21 participants in cohort 1. Over the 6-week intervention period, the average number of unique questions participants were enrolled in was 9.72 (SD 4.62; range 3-20). A total of 9 participants in design cycle 1 responded to the postprogram survey (Table 4). The majority of respondents, 6 (67%), indicated that they completed the cases during personal time, after work, or on weekends; 2 (22%) respondents indicated they completed the cases when they had time during the workday; and the remaining 1 (11%) participant completed the program while commuting.

### Design Cycle 2

There were 36 participants in cohort 2. Over the 6-week intervention period, the average number of unique questions participants were enrolled in was 9.69 (SD 5.79; range 1-22). A total of 15 participants in cohort 2 responded to the postprogram survey, but only 10 responded to the whole survey (Table 4). Of the 14 respondents who provided feedback on when they completed the program, the majority of respondents, 9 (64%), indicated they completed the cases during personal time, after work, or on weekends. In addition, 3 (21%) respondents indicated they responded to a question as soon as they received an alert, 11 (7%) respondents indicated they completed the cases when they had time during the workday, and the remaining 11 (7%) participants completed the program while commuting.

### Design Cycle 3

There were 18 participants in cohort 3. Over the intervention period, the average number of unique questions participants were enrolled in was 6.27 (SD 4.93; range 1-17). One participant responded to the postprogram survey for cohort 3 (Table 4). The respondent indicated they had responded to the questions during personal time, after work, or on weekends.

### Problems Encountered by Participants That Could Trigger a Question

Over the intervention period, EMR data indicated participants had a total of 2961 patient presentations that could have triggered a question. This number included duplicate instances of a participant seeing the patient presentation categories that could trigger a case. Of these, the most common presenting problem encountered by participants was abdominal pain (357 presentations), followed by mental health (159 presentations), and shortness of breath (156 presentations). Of the top 20 presenting problems participants encountered during the intervention period, all 7 presenting problem clusters that could have triggered a question in the online program were present (Figure 2).

## Discussion

### Principal Findings

Findings from this study indicate that EMR data can be used to personalize an online program for early-career physicians on management of common emergency presentations and link clinical practice directly with education in a timely manner. The researchers were able to extract EMR data at regular intervals during the intervention and use it to populate the adaptive algorithm that delivered personalized questions for individual participants. Regarding acceptability, findings suggested most participants found the program engaging and felt there was a level of alignment with their clinical practice. In addition, study findings indicated that early-career physicians undertaking intern training in emergency departments would like to see online programs personalized using electronic data in the future. This finding aligns with the literature, which has shown that health care providers across a range of professions are interested in seeing EMR data used in education and training [20].

### Insights on EMR Data for Enabling Adaptive Learning

The potential value of using analytics related to interactions with training programs to personalize learning has been well researched in the context of learning analytics [14]. In the context of medical education, harnessing practice data about learners in digital and online education has been noted as a means to improve evidence-based instruction [7], but there are still significant gaps in our understanding of how to do this. This study presents one of the first studies demonstrating how routinely collected EMR data can be used to personalize learning. This study demonstrated that in the context of medical practitioner education, there are unique opportunities to strengthen training using analytics of data from clinical sources such as EMRs, not just learner data generated by undertaking educational interventions [15].

In the context of this study, the course curriculum was developed by first working with domain experts to identify common clinical presentations that were challenging for participants, and then refining it based on what was feasible to extract from the EMR during the intervention. Retrospective analysis of EMR data extracted during the intervention highlighted that participants had commonly encountered a number of presenting problems that were not included in the curriculum (falls, back pain, and cough). An alternative approach involving data mining of EMR data to generate a preliminary curriculum, followed by domain expert review, may have strengthened the intervention. EMR data mining has not been used in this way to date, though the approach has been shown to have some use to identify areas where medical practitioners are deviating from best practice and may benefit from undertaking specific training [28].

Furthermore, findings from this study indicated that, while data can be used to personalize training, the quantity of data collected by EMR datasets is substantial, and choosing which data to use to prompt learning can be complex. A confounding factor is that while there is an upward rise for EMR adoption in health care globally [17], real-world adoption varies considerably across countries and health care settings. This may limit the availability of EMR data for supporting the type of intervention, but there are many other clinical information systems in use in the health system [29]. Data from different clinical information has been shown to have value for understanding aspects of practitioner performance [30] and may be able to be used for this type of adaptive intervention.

### Data-Driven Learning and Learner Engagement

Although it is feasible to adaptively deliver training using EMR data, findings from this study do not resolve how such an approach improves engagement with educational offerings. While study participants reported generally positive experiences with the program, there was also a notable decline in participation between the first and third cohorts. It is not clear why this decline occurred, but one explanation is that it aligned with the progression of the year. It is possible that participants undertaking the study were experiencing more exhaustion and had less capacity to engage with the intervention further along in their training year. There is some evidence to suggest that internship burnout is higher later in the year when completing postgraduate training [31].

The majority of learners in the personalized program attempted at least 50% of the program, which demonstrates a retention rate on the higher end for an online program [9]. Findings further indicated that many participants felt the intervention seemed aligned with their clinical practice. Aligning training with clinical practice may have a range of benefits for learners including increased engagement with training, better alignment with clinical practice, and more efficient delivery. Improving efficiency is important in medical practitioner education because learners are time-limited and have many competing obligations in their workloads beyond undertaking training, including completing core clinical responsibilities and administrative tasks [2,3].

### Strengths, Limitations, and Future Research

A limitation of this study is that the survey response rate across all three cycles was fairly low, particularly for the third cycle. This limits our overall understanding of the intervention and its generalizability. The lack of data on the final cycle limits understanding of how changing the content may have altered the learner experience with the program in the last cycle. Although we can only speculate as to why the response rate was low, one speculation is that the evaluation was done very late in the participants’ clinical term, and in some instances when they had started a new term. At this point in the term, participants had particularly busy clinical loads and may have had less capacity to provide feedback on the intervention.

Future researchers should consider further exploring how to design medical practitioner learning to be pedagogically sound and well-aligned with clinical practice. Design considerations that remain to be explored include developing a process for automated extraction of data to more closely link delivery of learning with the clinical encounter, identifying where in workflows adaptive training is optimally delivered (at point of care and after hours), and customizing content of the learning not just delivery using EMR data. It would be valuable to undertake studies that evaluate whether personalization of training using EMR data and the timing of content delivery affect learner retention rates, as well as improve the processes and outcomes of care. Beyond this, questions remain about the scalability and cost-effectiveness of adaptive learning interventions of this nature.

### Conclusions

This study demonstrates that personalizing an online learning program for emergency trainees using EMR data is feasible for this group of medical practitioners, and most also found it to be an acceptable approach to align learning with workplace interactions. This opens up considerable opportunity to tailor and personalize learning for health professionals that is aligned with their practice activities and performance. However, more research is needed to understand how to deliver these types of adaptive learning interventions in a scalable and sustainable way. To do this, there is a need to develop a better understanding of how routinely collected data can be used to understand performance, as well as its suitability enabling health professionals to reflect on their performance and practice to support continuous learning. Relatedly, on the solution design side, there is a need to develop platforms that can automate the extraction, analysis, and feedback of these data to support health professional education and practice reflection in a way that is streamlined for use by health professionals. Considerable focus to date has been placed on the value of aggregating large data sets into single repositories, which represents a significant infrastructure achievement. However, moving forward, it is as important to understand why data are being collected and how they will be collected to ensure that the right information is available to provide benefits to the health system and support people’s health and well-being.

## Supplementary material

10.2196/65287 Multimedia Appendix 1 An example of one of the cases used within the program, and images showing how the content was presented by the Qstream platform.

## Abbreviations

EMR: electronic medical record
HIT: health information technology
