# Supplementary material for: Using Electronic Health Data to Deliver an Adaptive Online Learning Solution to Emergency Trainees: Mixed Methods Pilot Study
Source: JMIR Med Educ. 2025 Dec 17;11:e65287. doi: 10.2196/65287 (PMC12711133; doi:10.2196/65287)

SYNCOPE CASE EXAMPLE

Meds mix up
Lola is a 68 year old female who has recently undergone a hip replacement and has represented to the Emergency Department (ED) from the local rehabilitation hospital with an episode of abdominal pain, vomiting and syncope. She remains nauseous in the ED ambulance bay.

Her previous medication list includes the following: atorvastatin 40 mg daily, ramipril 5mg daily, levodopa/carbidopa 100/25 1 tablet three times a day and entacapone 200 mg three times a day.

Which antiemetic medication would you prescribe?

a) (CORRECT) Domperidone 10mg orally three times a day prn
b) Metoclopramide 10mg orally three times a day prn
c) Prochlorperazine 5mg three times a day prn
d) None of the above

Take Home Message:
Metoclopramide and prochlorperazine both worsen Parkinson's disease symptoms, and therefore are contraindicated in those with Parkinson's disease. Domperidone is more appropriate in these patients.

Further Explanation:
The reasons for admission to hospital for patients with Parkinson's disease include infection, trauma, cardiovascular emergencies, cerebrovascular emergencies, gastrointestinal emergencies, and electrolyte disturbances. Antiemetics can be prescribed but we should avoid dopamine antagonists.

In any patient presenting with dizziness and/or syncope care must be taken in both assessment and management. From an assessment perspective, history and examination may be limited by the environment (e.g. the ED ambulance bay). Where communication is suboptimal, a collateral history is often useful. A number of life-threatening diagnoses should be considered in this case including abdominal (e.g. ischaemic gut, AAA), cardiovascular (e.g. myocardial infarction, aortic dissection), pulmonary (e.g. PE), autonomic and neurological.

The question asked highlights the issue with management of patients with Parkinson's on the one hand we must avoid medications that may worsen symptoms, on the other we must ensure that patients receive their regular medications avoid an exacerbation of their neurological disorder.

Patients with chronic disease often have a protracted stay in the ED. In these challenging cases UK doctor Mike Clancy offers a useful cognitive checklist to ensure a rigorous patient assessment:

COGNITIVE CHECKLIST

A. Are there "must not miss" diagnoses?

B. Did I stop thinking?

- Did I just accept the first diagnosis that came to mind?
- Was the diagnosis suggested to me by another?
- Was this patient handed over to me?
- Are there data that I haven't reviewed?

C. Did something stop my thinking?

- Was I interrupted, distracted or overloaded when evaluating this patient?
- Do I dislike this patient or like this patient too much or know them personally?

References:

1. Australian Medicines Handbook 2017
2. ACI Factsheet on Parkinson's Disease - aci.health.nsw.gov.au/__data/assets/pdf_file/0020/273503/pd-pathway-hornsby-2013.pdf
3. Cognitive errors - http://ragepodcast.com/tag/cognitive-debiasing

CASE EXAMPLE IN QSTREAM APP

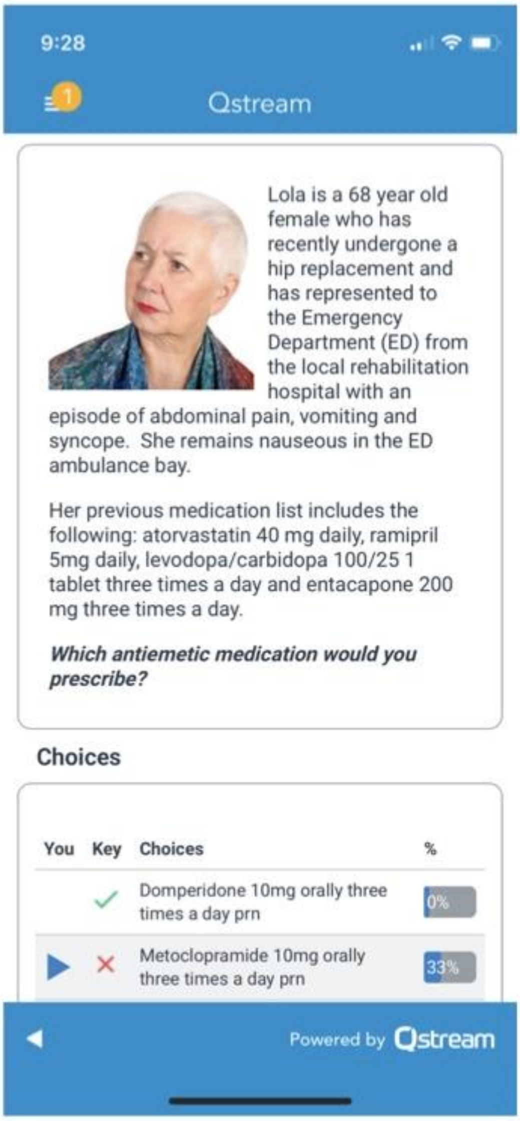

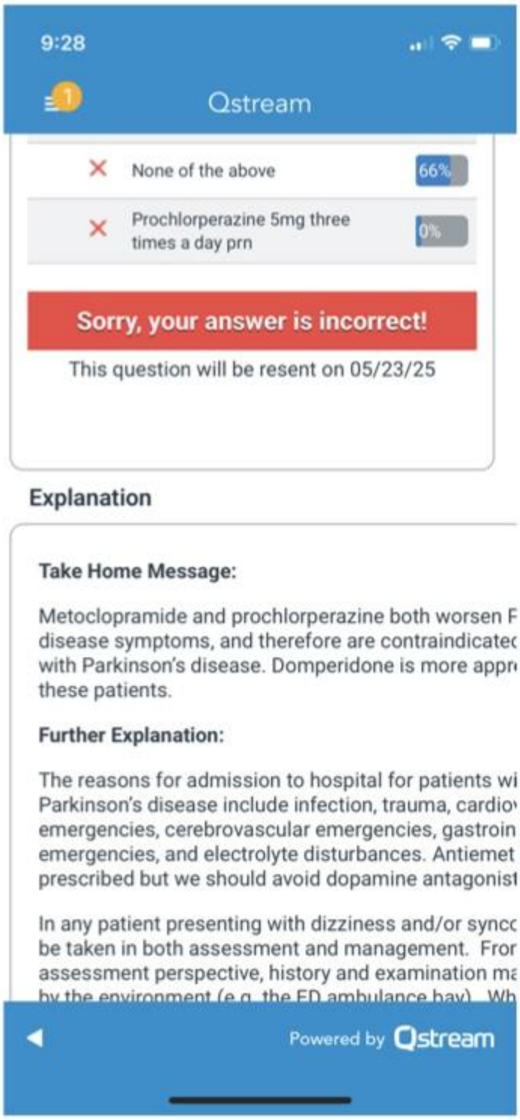

Supplement: Multimedia Appendix 1 [file mededu-v11-e65287-s001.pdf]
